# Editorial: The influence of lifestyle factors on cancer biology and treatment efficacy

**DOI:** 10.3389/fphys.2023.1254151

**Published:** 2023-07-31

**Authors:** Mark D. Ross, James E. Turner, Samuel T. Orange, Richard S. Metcalfe

**Affiliations:** ^1^ School of Energy Geoscience, Infrastructure and Society, Institute of Life and Earth Sciences, Heriot-Watt University, Edinburgh, United Kingdom; ^2^ School of Sport, Exercise and Rehabilitation Sciences, University of Birmingham, Birmingham, United Kingdom; ^3^ School of Biomedical, Nutritional and Sport Sciences, Faculty of Medical Sciences, Newcastle University, Newcastle, United Kingdom; ^4^ Applied Sports Technology, Exercise and Medicine Research Centre, Swansea University, Swansea, United Kingdom

**Keywords:** cancer, lifestyle, diet, exercise, chemotherapy, physical activity, cardiotoxicity

In 2019, 23.6 million global cancer cases and 10 million deaths related to cancer were recorded. These statistics have increased by 26.3% and 20.9%, respectively, since 2010 (Global Burden of Disease 2019 Cancer Collaboration et al., 2022). By 2040, it is estimated that there will be 27.5 million global cancer cases and 16.3 million deaths, due to an ageing population and increasing prevalence of risk factors, including physical inactivity and obesity (Bray et al., 2018). It is established that a physically active lifestyle reduces cancer risk (Moore et al., 2016), but accumulating evidence shows that being active after a cancer diagnosis, and during and after treatment, is associated with positive clinical outcomes (Christensen et al., 2018). This Research Topic presents a collection of original cancer-related articles in epidemiology, immunology, cardiovascular physiology, and a mini-review on musculoskeletal health in cancer settings.

Metabolic syndrome is associated with an increased risk of colorectal cancer (CRC), but the modifying effect of healthy lifestyle behaviours is unknown. Examining data from 328,000 UK participants followed up for a median of 12.5 years, Xie et al. assessed the individual and combined effects of metabolic syndrome and healthy lifestyle behaviours on the risk of colorectal cancer incidence and mortality. A healthy lifestyle score was derived from four modifiable behaviours (smoking, alcohol consumption, diet, and physical activity) and categorised as “favourable,” “intermediate,” or “unfavourable.” The presence of metabolic syndrome was associated with a 24% increased risk of both CRC incidence and mortality. Participants with metabolic syndrome and an unfavourable lifestyle had a 1.5–2-fold increased risk of CRC incidence and mortality compared to those without metabolic syndrome and a favourable lifestyle. The data suggest that adopting healthy lifestyle behaviours, even in the presence of metabolic syndrome, can help reduce the risk of CRC incidence and mortality.

People living with and beyond cancer often exhibit accelerated reductions in muscle strength and mass, which are associated with poor treatment tolerability and worse survival (Dunne et al., 2023). Currently, evidence supporting the utility of exercise interventions to improve muscle mass during cancer treatment is unconvincing (Clifford et al., 2021). In their mini-review, Brooks et al. recommend four key considerations when designing intervention trials targeted at improving muscle mass and strength in people living with and beyond cancer, namely, 1) define the condition of interest (i.e., sarcopenia, frailty, and/or cachexia); 2) determine the most appropriate outcome to evaluate intervention success; 3) establish the best timepoint to intervene to optimise outcomes, and 4) create evidence-based recommendations for how exercise prescription can be configured to optimise outcomes. More high-quality evidence in this area has potential to improve clinical outcomes, particularly in cancer types characterised by the loss of muscle mass.

Studies comparing leukocyte characteristics between cancer survivors and non-cancer controls are often interpreted as providing evidence of disease and/or treatment impacting immunity, but most studies do not account for the influencing effects of participant characteristics or their lifestyle. Filling this gap, Arana Echarri et al. compared sub-populations of T cells between healthy women (*n* = 38) and breast cancer survivors (*n* = 27) within 2 years of treatment. Survivors exhibited signs of immunosenescence (e.g., lower CD8^+^ naïve T-cell counts), and across CD4^+^/CD8^+^ effector/memory T-cell sub-types, the proportion of activated (HLA-DR+) cells was higher than that in healthy women. Uniquely, Arana Echarri et al. reported divergent moderating effects of age, cardiorespiratory fitness, body composition, and cytomegalovirus serostatus depending on the cell type and variable examined. For the first time, Arana Echarri et al. showed that the fat mass index was positively associated with the proportion of activated effector/memory T cells, which withstood statistical adjustment for all other participant characteristics and lifestyle factors, implicating these cells as contributors to inflammatory/immune dysfunction in overweight/obesity.

Chemotherapy treatments are toxic to cardiovascular tissues, and the type and extent of the toxicity is dependent on the regimen (Yeh and Bickford, 2009). Hypertension is a common side effect of chemotherapy, likely due, in part, to endothelial dysfunction (Hader et al., 2019). Thus, interventions are being sought to alleviate, or attenuate, the detrimental effects of chemotherapy on the cardiovascular system. Mclaughlin et al. developed a serological method to determine whether exercise training can protect against endothelial cell apoptosis induced by common breast cancer chemotherapies. Prior to exposure to 5-fluorouracil, epirubicin, cyclophosphamide (FEC), and docetaxel, human coronary artery endothelial cells (HCAEC) were incubated with serum collected before and after a 12-week exercise training intervention in 12 women. Chemotherapy, *in vitro*, caused endothelial cell apoptosis in a dose-dependent manner. Endothelial cell wound healing was also inhibited. Prior incubation with post-exercise training serum resulted in reduced apoptosis in response to FEC and improved wound healing after being exposed to docetaxel (both vs. serum from pre-exercise training).

Exercise has potential to modulate the tumour immune microenvironment. In rodents, voluntary wheel running results in tumour growth reduction, due to infiltration of natural killer (NK) cells (Pedersen et al., 2016). Gupta et al. investigated whether different exercise training protocols during gemcitabine treatment affected pancreatic tumour growth and tumour-infiltrating lymphocytes. Mice were assigned to either low- or high-volume continuous exercise, high-intensity interval exercise, or control groups. Among mice treated with gemcitabine, tumour volume increased within 20 days of inoculation with no effect of exercise. In mice without gemcitabine, the data trend demonstrated that mice undertaking low-volume continuous exercise had attenuated tumour growth. These effects were accompanied by no differences in tumour lymphocyte infiltrates. Currently, human studies support these findings, with no influence of exercise training on tumour infiltrates or tumour size among men with prostate cancer (Schenk et al., 2022; Djurhuus et al., 2023).

This Research Topic provides important advances in “lifestyle oncology,” but many questions remain unanswered. Bringing together exercise scientists, cell and molecular biologists, nutritionists, and clinicians/oncologists to share expertise and guide the future research will yield new advances.
